# A scoping review of traditional food security in Alaska

**DOI:** 10.1080/22423982.2017.1419678

**Published:** 2018-01-05

**Authors:** Amanda Walch, Andrea Bersamin, Philip Loring, Rhonda Johnson, Melissa Tholl

**Affiliations:** ^a^ Department of Biology & Wildlife, University of Alaska Fairbanks, Fairbanks, AK, USA; ^b^ School of Environment and Sustainability, University of Saskatchewan, Saskatoon, Saskatchewan; ^c^ Department of Health Sciences, University of Alaska Anchorage, Anchorage, AK, USA; ^d^ Department of Dietetics & Nutrition, University of Alaska Anchorage, Anchorage, AK, USA

**Keywords:** Traditional foods, food security, food access, food availability, food utilisation, Alaska native, indigenous

## Abstract

Food insecurity is a public health concern. Food security includes the pillars of food access, availability and utilisation. For some indigenous peoples, this may also include traditional foods. To conduct a scoping review on traditional foods and food security in Alaska. Google Scholar and the High North Research Documents were used to search for relevant primary research using the following terms: “traditional foods”, “food security”, “access”, “availability”, “utilisation”, “Alaska”, “Alaska Native” and “indigenous”. Twenty four articles from Google Scholar and four articles from the High North Research Documents were selected. The articles revealed three types of research approaches, those that quantified traditional food intake (n=18), those that quantified food security (n=2), and qualitative articles that addressed at least one pillar of food security (n=8). Limited primary research is available on food security in Alaskan. Few studies directly measure food security while most provide a review of food security factors. Research investigating dietary intake of traditional foods is more prevalent, though many differences exist among participant age groups and geographical areas. Future research should include direct measurements of traditional food intake and food security to provide a more complete picture of traditional food security in Alaska.

## Introduction

Food insecurity is a growing public health concern in Alaska, where over 13% of individuals or households are food insecure [1]. In rural communities and among Alaska Native people, the prevalence is even higher [2]. Distinct from most of the USA, the food system in Alaska is based on both commercial (or “market”) and subsistence food. Subsistence foods, also called “traditional”, “country” or “wild” foods, play a pivotal role in the health and food security of communities in Alaska. An estimated 65% of Alaska residents practice some form of subsistence activities and subsistence foods contribute upwards of 50% of average energy intake, depending on the region [2–6]. Subsistence foods are particularly important in rural Alaskan communities. The Alaska Department of Fish and Game estimates that the annual harvest of subsistence foods in rural communities averages 295 pounds per person, compared to 22 pounds per person in urban areas [7].

A growing understanding of the role traditional, culturally appropriate foods play in the health and well-being of Indigenous peoples has driven an increased global interest in expanding the definition of food security. Food security is often described as resting on three pillars: availability, access and utilisation. Availability is having sufficient quantities of food on a consistent basis, access includes the ability to purchase food or attain food from other sources and utilisation is the ability to meet daily nutrient requirements [8]. In the context of Indigenous peoples, these pillars should pay particular attention to traditional foods, which are meaningful in various pedagogical, psychological, cultural and social ways [9–14]. This focus on traditional foods when considering food security is what we call “traditional food security” and can, at a general level, only be defined loosely, given that food takes on different meanings for different people in different places. Specific definitions must therefore start with communities themselves, such as groundbreaking work by the Inuit Circumpolar Council, Alaska, on this topic [12]. Additionally, the concept of traditional food security incorporates the political and human ecologies of food. In the North, for example, it is well known that subsistence or “country” foods are essential aspects of local well-being and community sustainability; not surprisingly, high and rising rates of food insecurity, for example in northern Alaska and northern Canada, are widely considered threats to both physical health and cultural survival [12,15]. Traditional foodways are also an important way that many place-based cultures have forged reciprocal, mutually beneficial relationships with their local land and seascapes [11,16]. Thus, the concept of traditional food security may be an effective bridging concept between traditional food security research, which often focuses largely on assessment and intervention [17], and emerging community development discourses on food sovereignty, which starts explicitly from the problematisation of the power relationships inherent in contemporary food systems [11].

There are some emerging and conflicting ideas about the state of traditional food security in rural Alaska. Community patterns differ with respect to the extent of the resilience and sustainability of traditional foodways, including the role of a mixed cash and subsistence economy, and whether all people’s cultural, spiritual and nutritive needs are being met through the current traditional food system [12,18,19]. What is clear is that food insecurity remains a problem for many; and regardless of these apparently shifting trends, when someone is food insecure, it impacts emotional, social and physical health.

Understanding the prevalence of traditional food insecurity and its causes and consequences in Alaska is relevant to public health professionals, food assistance programmes, policymakers and tribal leaders and may ultimately be a key to improving Alaska Native peoples’ health. Synthesis of primary research evidence about traditional food security is needed to better determine what is already known about food insecurity, who is at risk for food insecurity, why they are at risk and for developing strategies and interventions to improve food security [17]. Thus, the objectives of this scoping review were to (1) review primary research to better understand what is known about food security in Alaska; (2) evaluate what is known about the causes and consequences of traditional food insecurity in Alaska, operationalised as paying-specific attention to how the three pillars of food security apply to traditional foods and (3) discuss research gaps and future research needed in the field.

**Figure 1.**
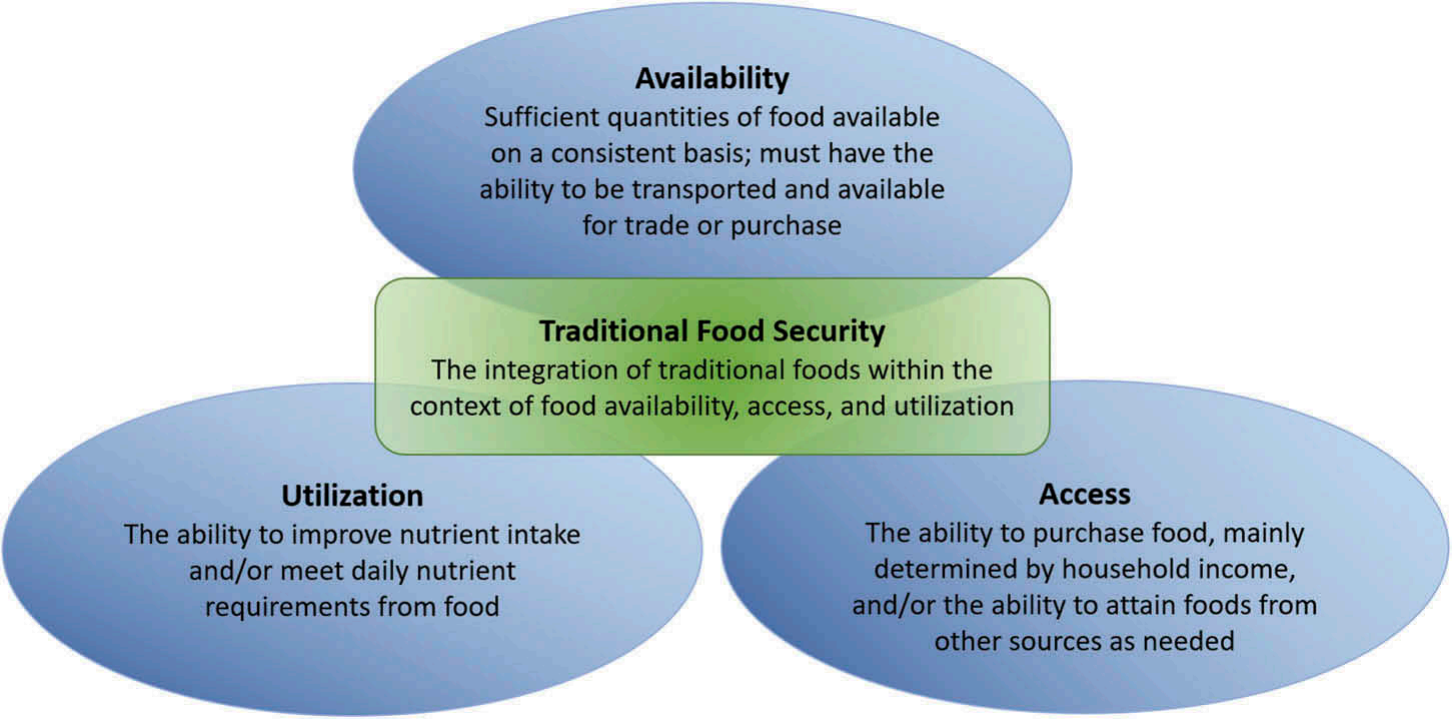
Pillars of food security.

Figure 1 describes the pillars of food security [8,18].

## Methods

### Data sources

In September 2015, peer-reviewed primary research articles were identified using two online databases: Google Scholar and the High North Research Documents. The search aimed to identify qualitative or quantitative studies addressing traditional food intake and/or one or more pillars of food security. Keywords for our initial search included the following: traditional foods, food security, food access, food availability, food utilisation, Alaska, Alaska Native and indigenous.

### Study inclusion and exclusion criteria

Article abstracts had to meet the following criteria for inclusion in the review: (1) written in English, (2) primary quantitative or qualitative research that addressed at least one pillar of food security and/or traditional food intake, (3) inclusion of human research subjects and (4) location in Alaska. Articles that did not address food security or traditional food intake were review articles, grey literature, and articles that reported on “local” food rather than “traditional” foods were excluded from the review.

### Analysis

Studies within the scoping review were categorised into three main types: (1) those that quantified traditional food intake, (2) those that quantified food security and (3) qualitative articles that addressed at least one pillar of food security. Research that quantified traditional food intake focused on understanding how traditional foods contribute to nutrient intake or diet quality. Research quantifying food security included estimates of food security/insecurity. Qualitative studies examined participant observations or perspectives on factors impacting food security. The three categories were used to evaluate how traditional foods relate to the pillars of food security in Alaska, to identify gaps in current knowledge and to determine future research needs.

## Results

The initial search on Google Scholar yielded 54,700 hits. Abstracts were scrutinised for inclusion criteria, starting with the most relevant hits. After reviewing the first 100 articles, no further research meeting inclusion criteria were found. Of the 100 articles examined, 24 met the inclusion criteria and were selected for the review. The High North Research Documents database yielded 3,264 initial hits with filters for Alaska and papers written in English. Abstracts of the first 80 documents were reviewed; it was determined that four articles met inclusion criteria and were identified for the review. In total, 28 articles were included in this review.

Table 1 summarises the results by the type of research, geographic location and study population, if diet was measured and how, if food security was measured and how, and the pillar of food security addressed. These categories were selected to highlight key variables under study and to better understand gaps in the literature on traditional food security in Alaska.

**Table 1.** 

Article(s) and study name (if applicable)	Geographic location	Study population	Diet measured? How?	Food security measured? How?	Food security pillar
Quantitative traditional food intake articles					
Ballew et al. [3] The Alaska Traditional Diet Survey	13 Remote villages statewide	Alaska Native women (n=401) and men (n=253) aged 13–88 years	Yes – interviewer administered FFQ with portion sizes over the past 12 months	No	Food access and food utilisation
Bartell et al. [22] What People Eat: atka, Alaska, 1998–1999	1 Remote community in the Aleutians	34 of the 80 residents in Atka of unknown ages	Yes – FFQ and a 24-h recall	No	Food utilisation
Bersamin et al. [23] The Center for Alaska Native Health Research Pilot Study	3 Remote communities in SW Alaska	Alaska Native women (n=44) and men (n=48), aged 14–81 years	Yes – single 24-h recall	No	Food utilisation
Bersamin et al. [4] The Center for Alaska Native Health Research Study	7 Remote communities in SW Alaska	Alaska Native women (n=307) and men (n=241), aged 14–94 years	Yes – single 24-h recall	No	Food utilisation
Bersamin et al. [43] The Center for Alaska Native Health Research Study	7 Remote communities in SW Alaska	Alaska Native women (n=301) and men (n=230), aged 14–94 years	Yes – 24-h recall and a 3-day food from 54% of the participants	No	Food utilisation
Eliat-Adar et al. [44] Genetics of Coronary Artery Disease in Alaska Natives (GOCADAN) Stud	7 Remote villages in the Norton Sound region	Alaska Native women (n=677) and men (n=537), aged 18 years and older	Yes – FFQ from previous year; 97 food items classified into 28 food groups	No	Food utilisation
Heller and Scott [20] The Alaska Dietary Survey: 1956–1961	11 Rural villages in Alaska	Alaska Native women and men, children and adults	Yes – diet records of 3–7 day duration; collected on a seasonal basis	No	Food utilisation
Johnson et al. [21] The Alaska Native Dietary and Subsistence Food Assessment Project	12 Remote communities in SW Alaska	Alaska Native women (n=218) and men (n=115), aged 13–88 years	Yes – 24-h recalls during four seasons	No	Food utilisation
Luick et al. [41] The Center for Alaska Native Health Research Study	7 Remote communities in SW Alaska	Alaska Native women (n=284) and men (n=213), aged 14 years and older	Yes – 24-h recall and a subsample with an additional 3-day diet record	No	Food utilisation
Nash et al. [42] The Center for Alaska Native Health Research study	7 Remote communities in SW Alaska	Alaska Native women (n=403) and men (n=350), aged 18–94 years of age and older	Yes – 24-h recall and a 3-day diet record	No	Food utilisation
Nobmann et al. [28] The diet of Alaska Native Adults: 1987–1988	11 Urban and remote communities	Alaska Native women (n=186) and men (n=165), aged 20–63 years	Yes – 24-h recalls during 5 seasons over an 18-month period	No	Food utilisation
Nobmann et al. [26] The Alaska Siberia Project	1 Remote community (Gambell, Alaska)	Alaska Native women (n=57) and men (n=70), aged 40–87	Yes – 24-h dietary recall and a semi quantitative FFQ from the past year	No	Food utilisation
Nobmann and Lanier [24]	1 Urban community	Alaska Native women (n=74), aged 18–45 years	Yes – 24-h recalls (goal of 4)	No	Food utilisation and food access
Nobmann et al. [6] Genetics of Coronary Artery Disease in Alaska Natives (GOCADAN) Study	7 Remote villages in the Norton Sound region	Alaska Native women (n=480) and men (n=370), aged 17–92 years	Yes – semi-quantitative FFQ	No	Food utilisation and food access
Redwood et al. [37] The Education and Research Towards Health (EARTH) Study	26 Urban and remote communities	American Indian and Alaska Native women (n=2,323) and men (n=1,507), aged 18 and older	Yes – A semi quantitative diet history questionnaire tailored to region	No	Food access
Risica et al. [27] The Alaska Siberia Project	4 Remote communities in NW Alaska	Alaska Native women (n=225) and men (n=209), aged 25 or older	Yes – 24-h dietary recall	No	Food utilisation
Sharma et al. [25] The Alaska Native Dietary and Subsistence Food Assessment Project	6 Remote communities in SW Alaska	Alaska Native (Yup’ik) women, non pregnant or lactating (n=82), aged 18 and over	Yes – up to three 24-h recalls	No	Food utilisation
Smith et al. [30] The Alaska WIC Healthy Moms Survey *Also listed in Quantitative Food Security Articles	5 Rural and 1 urban communities	Predominantly (64%) Alaska Native women (n=122;: 60 from rural Alaska and 62 from urban Alaska), aged 19 and older	Yes – structured nutrition interviews (24-h recall and the Block Brief Food Frequency tool)	Yes – doesn’t state	Food access and food utilisation
Quantitative Food Security Articles					
Loring et al. [29]	The Kenai Peninsula region	490 Randomly selected households	No	Yes – 6 question “coping strategies” questionnaire	Food access
Smith et al. [30] The Alaska WIC Healthy Moms Survey *Also listed in Quantitative Traditional Food Intake Articles	5 Rural and 1 urban communities	Predominantly (64%) Alaska Native women (n=122; 60 from rural Alaska and 62 from urban Alaska), aged 19 and older	Yes – structured nutrition interviews (24-h recall and the Block Brief Food Frequency tool)	Yes – doesn’t state	Food access and food utilisation
Qualitative Food Security Articles					
Brubaker et al. [31]	12 Remote communities in NW Alaska	Communities were predominantly (85%) Alaska Native Inupiat Eskimos	No	No	Food utilisation
Burke et al. [39]	9 Remote communities in Southcentral and SE Alaska	Predominantly Caucasian women (n=23) and men (n=11)	No	No	Food access
Fazzino and Loring [35]	1 Urban community	Alaska Native men and women (n=39)	No	No	Food access
Flint et al. [32]	3 Remote communities in southcentral Alaska, the E Aleutian Islands, and NW Alaska	Predominantly Alaska Native men and women from 65 households	No	No	Food utilisation and food utilisation
Hupp et al. [40]	73 Remote and urban communities statewide	96 Individuals, both men and women	No	No	Food availability
Loring and Gerlach [33]	6 Remote communities along the Yukon River	25 Alaska Native individuals (predominantly men), aged 40–90 years	No	No	Food access and food availability
Magdanz et al. [36]	6 Remote NW Alaska communities	92 Alaska Native households	No	No	Food availability and food access
McNeeley and Shulski [34]	3 Remote communities in SW Alaska	25 Alaska Native individuals, aged 55 and older	No	No	Food availability and food utilisation

### Type of research

The majority of the studies were quantitative (n=19). Of these, 18 focused on estimating traditional food intake and 2 estimated food security (1 of which also estimated traditional food intake). Eight studies were qualitative and addressed at least one pillar of food security.

A majority (n=13) of the research that estimated traditional food intake found that people who consumed traditional foods had higher intakes of several important nutrients, including protein, omega-3 fatty acids and iron [3,4,20,21]. Other articles compared the dietary intake of people who consumed traditional foods against a national sample or national recommendations [20,22–25] and in many situations concluded that traditional foods help to meet these recommendations, especially for energy, protein and fat intake (n=9) [21,25–27]. Several studies also found that among people consuming traditional foods, recommendations were not met for other key nutrients, including calcium and fibre [20–22,28].

Two articles were found that quantified food security, though neither measured food security using the USDA’s Adult Food Security Survey Module. One formulated a “coping strategies” questionnaire to measure food security and the other article did not state the tool used to measure food security. The studies found varying food insecurity rates, ranging from 7% to 27% in urban settings and up to 39% in rural settings [29,30].

The eight qualitative studies examined peoples’ perspectives on factors impacting food security. Participants identified climate change [31,32], concerns about contamination in the traditional food supply [32] and changing wildlife migration patterns [33,34] as causes of traditional food insecurity. Additional factors included high equipment and fuel prices [35,36] and a loss of traditional knowledge [32].

### Geographic location

Research on traditional food intake was broadly distributed across the state, although the majority (n=24) were conducted primarily in rural areas. One was conducted exclusively in an urban area [24] and three others were conducted in both urban and rural areas [28,30,37]. Participants who were interviewed came from communities of different sizes and locations, with varying levels of road system access (Table 1).

Two studies estimated food security, one of which was conducted in an urban community of approximately 55,000 people [29]. The second study was conducted in both an urban area (population 30,000) and rural area [30]. No studies measured food security exclusively in rural areas where food insecurity has been reported to be the highest [38] (Table 1).

Of the eight qualitative food security studies, one was conducted in an urban area [34] and those remaining seven were conducted in rural communities (Table 1).

### Study population

The study populations in this scoping review included a range of ages and ethnic groups. All the research estimating traditional food intake were conducted among Alaska Native women and men. Of those, 10 focused on adults only while 7 included adolescents as young as 13 in addition to adults. One study included all age groups but did not specify how many and of what age [20]. No studies estimated traditional food intake among children only (Table 1).

Food security was measured among non-pregnant and non-breastfeeding adult women [30] and a random sampling of both women and men [29]; both samples included Alaska Native people.

Six qualitative studies were conducted predominantly among Alaska Native people. One study was conducted primarily among non-Alaska Native people [39] and one did not specify [32] (Table 1).

### Traditional food availability

No studies that quantified traditional food intake or food security addressed traditional food availability. Four of the qualitative articles addressed the availability of traditional foods. In particular, research found that climate change may limit the availability of traditional foods due to the effect of warmer and/or unpredictable temperatures on game mating and hunting timing [34] and changing migration patterns of some fish species [33,36]. Additionally, it was observed that the abundance of wild berries has declined or become more variable in the past decade [40] (Table 1).

### Traditional food access

Fourteen articles looked at traditional food access and concluded that traditional foods are less readily accessible in urban areas where a majority of the indigenous population are now living [24] and where preference for these foods is declining, especially among younger generations [6]. Both the qualitative and quantitative food security articles show that access to traditional foods, in both urban and rural areas, is increased when individuals share or trade traditional knowledge to harvest and prepare traditional foods, share the equipment needed to attain the foods, or share or trade the actual foods [29,30,35,39]. Several of the articles mentioned that access to traditional foods requires consideration of the costs of hunting and fishing equipment and fuel needed for transportation to hunt, fish or gather [35,36] (Table 1).

### Traditional food utilisation

Nineteen articles looked at traditional food utilisation. There were two qualitative food security studies that reviewed factors causing traditional food spoilage or food-borne illness. One reported that climate change and warming temperatures are thawing underground food storage cellars and causing food safety issues [31]. The second article indicated that traditional foods are spoiling due to the length of time required to go from the changing migration grounds, which are farther away, to the processing locations [34] (Table 1).

The majority of the papers (n=14) focused on the nutrient quality and health benefits of traditional foods. Individuals who consume traditional foods have high levels of many nutrients in their diet, including protein, vitamin A, most B vitamins, vitamin C, vitamin D, vitamin E, phosphorus, iron, niacin, selenium and omega-3 fatty acids [3,4,6,20,21,25,27,30,41]. The amount of traditional foods consumed varied by age, education and geographic location in the study populations [4,6,42]. Several articles concluded that traditional foods promote cardiovascular health [6,24,43,44] and a lower prevalence of glucose intolerance [21].

## Discussion

Though traditional food security has been loosely defined in the literature [13,14], it can be conceptualised as paying particular attention to traditional foods when considering food availability, access and utilisation. Studies that estimate the prevalence of food insecurity in remote Alaska Native communities, where poverty is pervasive and food availability is limited, are virtually absent from the literature. Using conventional measures, 14% of the overall population, at least 19% of the Alaska Native population, and 20–25% of rural communities are experiencing food insecurity [2,38]. Primary research is lacking to fully understand its causes and consequences, and the potentially vital role that traditional foods play in achieving food security. Of the 28 studies, only 2 quantify food security. Many Alaska Native peoples continue to consume a wide variety of traditional foods and these foods make up 15–22% of the diet in some rural communities [3,4,6]. It is unknown to what extent traditional foods contribute to the food security status of Alaska Native people since the role of traditional foods is still so poorly understood in relationship with food security.

Traditional foods are an important source of essential nutrients in rural Alaska Native communities. Consistent with research conducted in the Canadian Arctic, people who consume higher levels of traditional foods have higher intakes of protein, vitamin D, iron, omega-3 fatty acids and other vitamins and minerals. These patterns suggest that traditional foods are essential to maintaining adequate nutrition and enhancing both health and food security [45]. This is not surprising given that the food system in rural Alaska Native communities is dependent on both traditional foods and food imports that can be unreliable and of poor quality because of air or other transportation services that are routinely impacted by harsh and unpredictable environmental conditions.

Today more than half of Alaska Native people live in urban centres, where store-bought foods are readily available, while traditional foods are much harder to access [46]. Yet, to our knowledge, few studies have investigated the contribution of traditional foods to food security in urban areas. These include Fazzino and Loring [35] who focus specifically on the experiences of food bank users who have recently moved to urban areas from rural communities, and Harrison and Loring [47] who interviewed harvesters of salmon and clams in Cook Inlet. This research gap could be significant for understanding the health status of urban indigenous peoples, because as noted, the increased consumption of store-bought foods has been shown to decrease nutrient and diet quality and increase the potential for chronic diseases such as obesity, diabetes and heart disease in both Alaska and the Circumpolar North [4,48–50]. Conversely, an increased intake of traditional foods has been associated with better health outcomes such as improved glucose tolerance and lipid profiles [51], lower risk of cardiovascular disease [28] and higher diet quality [13,52]. A fuller understanding of the role traditional foods play for those living in urban communities will also support policies to increase availability and access of these foods, such as provision of subsidies to help secure traditional foods or explicit allowance of traditional foods in food assistance programs. Such policy initiatives could also serve to strengthen the food system of indigenous populations throughout the state [45,47].

Alaska might also consider other more comprehensive or innovative policy initiatives that strengthen traditional food security in the Arctic such as the recent Nunavut Food Security Strategy and Action Plan [53] or investigating the feasibility of the development of traditional food markets [54]. The Nunavut strategy includes guiding principles and the incorporation of six dimensions of food security: country/traditional food, store bought food, local food, life skills, program and community initiatives, and policy and legislation. As noted in one recent assessment to promote traditional Inuit food by pregnant women in Nunavik, it is important that policymakers encourage formal coordination guidelines and appointment of project coordinators for each key institution involved in collaborative public health initiatives to assure implementation fidelity across sites [55].

What we know about traditional food security in Alaska comes from few primary research studies that vary in their methods and objectives, making it difficult to acquire a comprehensive and comparable review of factors that influence access, availability and utilisation of traditional foods. Factors that appear to influence traditional food availability and access include climate change, food sharing, living in urban areas, costs associated with following a traditional lifestyle and changing food preferences. Although a broad range of research has been conducted on climate change in Alaska and potential contaminants in the traditional food supply, very few of the studies came up on the database search and because there typically was no direct focus on food security, they fell outside the scope of this review. Additional research about traditional food security in the Circumpolar North could help provide further insights since the diverse region has similarities in its extreme climate, limited infrastructure and indigenous populations, though comparative research to date is rare [17]. For example, there seem to be many factors in the Circumpolar North that are impacting traditional food availability and access, such as differing species or hunting locations, changing animal migration patterns [56] and regulations on wildlife management [57]. Other factors identified by those outside of Alaska are related to the environment and climate change, ranging from a rise in sea levels from melting ice caps and glaciers [58] to thawing permafrost [56] and changing weather and winds [59].

Currently, surveillance data are collected in Alaska using the USDA Food Security Survey Module to estimate the prevalence and severity of food insecurity [1]. However, this tool has been critiqued for not capturing issues relating to the availability and access of traditional foods derived from northern environments [60]. For example, the instrument emphasises cash for purchasing food as a primary determinant of access and also invokes the western notion of a “balanced” diet which may be interpreted quite differently in northern contexts. As such, this standard survey may not fully capture the unique situation of the indigenous populations in Alaska due to traditional food preferences or the seasonal nature of the regional foods system [61]. Some work has been done to blend these kinds of surveys with other approaches to dietary recall in Alaska and elsewhere [30,62,63], but these are exceptions that have not yet informed common practice. In a promising development, the Alaska branch of the Inuit Circumpolar Council has produced a new framework for evaluating traditional food security [12]. Ideally, a common tool for use with indigenous populations in both urban and rural communities could be developed to allow for comparison and to provide a more comprehensive understanding of the local causes and consequences of traditional food security in Alaska.

This scoping review is the first to our knowledge to systematically investigate the state of evidence regarding the contribution of traditional foods to food security of Alaska Native peoples. One potential limitation of this review was the exclusion of “local” foods research. This may have resulted in missed studies that could contribute to our understanding of how Alaskans meet food security needs outside of market foods purchased with cash but added clarity to our assessment of literature that focused on foods construed as “traditional”. Grey literature was not included in this review due to its typical absence within conventional databases. A determination was made to focus on primary research for this review in order to best complement and supplement the findings that are more easily available from other published reports, such as food security reports from the State of Alaska, Division of Subsistence.

### Conclusion

Food security is clearly an important public health issue for many Alaskans, yet there is insufficient meaningful primary research investigating unique Alaskan food security issues. In a state with vast geographical areas and extreme weather conditions, many rural Alaska Native communities and urban-dwelling Alaska Native individuals face challenges attaining adequate, safe and culturally appropriate foods. To further understand the causes and consequences of traditional food insecurity in Alaska, and to increase efforts necessary to promote traditional food security, future research should use a common tool to directly measure traditional food access and availability across all communities in the state.

Working with and across disciplines (and across arctic borders) will be necessary to aid in the efforts. Examples include working with legislators and other policymakers to write and pass applicable laws to promote traditional food security, developing culturally appropriate nutrition education to promote traditional foods for those receiving food and nutrition assistance and collaborating with circumpolar colleagues to design shared food-related studies that acknowledge and measure the unique contributions of traditional foods to better health and increased food security in Alaska.
