# Corrigendum

**DOI:** 10.1080/22423982.2018.1433615

**Published:** 2018-02-13

Walch A, Loring P, Johnson R, et al. A scoping review of traditional food security in Alaska. Int J Circumpolar Health 2017;77(1): 1419678.

https://doi.org/10.1080/22423982.2017.1419678

When this article originally published online, the structure of the abstract, corresponding author details and the order of the authors were incorrect. This information has now been corrected as shown below:

Amanda Walch^a^, Philip Loring^b^, Rhonda Johnson^c^, Melissa Tholl^d^ and Andrea Bersamin^e^

^a^Department of Biology & Wildlife, Center for Alaska Native Health Research, Institute of Arctic Biology, University of Alaska Fairbanks, Fairbanks, AK, USA;

^b^School of Environment and Sustainability, University of Saskatchewan, Saskatoon, Saskatchewan;

^c^Department of Health Sciences, University of Alaska Anchorage, Anchorage, AK, USA;

^d^Department of Dietetics & Nutrition, University of Alaska Anchorage, Anchorage, AK, USA;

^e^Center for Alaska Native Health Research, Institute of Arctic Biology, University of Alaska Fairbanks, Fairbanks, Alaska

**CONTACT** Andrea Bersamin abersamin@alaska.edu Center for Alaska Native Health Research, Institute of Arctic Biology, University of Alaska Fairbanks, Fairbanks, Alaska, USA

The abstract has been formatted as shown below:

**Background**: Food insecurity is a public health concern. The pillars of food security include food access, availability and utilisation. For some indigenous peoples, the pillars may focus on traditional foods.

**Objective**: To conduct a scoping review on traditional foods and food security in Alaska.

**Design**: Google Scholar and the High North Research Documents were used to search for relevant primary research using the following terms: “traditional foods”, “food security”, “access”, “availability”, “utilisation”, “Alaska”, “Alaska Native” and “indigenous”.

**Results**: Twenty four articles from Google Scholar and four articles from the High North Research Documents met the inclusion criteria. The articles revealed three types of research approaches, those that quantified traditional food intake (n=18), those that quantified food security (n=2), and qualitative articles that addressed at least one pillar of food security (n=8).

**Conclusions**: Studies that estimate the prevalence of traditional food insecurity in Alaska are virtually absent from the literature.  Instead most studies provide a review of factors related to food security. Research investigating dietary intake of traditional foods is more prevalent. Future research should include direct measurements of traditional food intake and food security to provide a more complete picture of traditional food security in Alaska.

